# Cognitive, Emotional and Psychological Manifestations in Amyotrophic Lateral Sclerosis at Baseline and Overtime: A Review

**DOI:** 10.3389/fnins.2019.00951

**Published:** 2019-09-10

**Authors:** Soumia Benbrika, Béatrice Desgranges, Francis Eustache, Fausto Viader

**Affiliations:** Neuropsychology and Imaging of Human Memory, Normandy University-PSL Research University-EPHE-INSERM U1077, Caen University Hospital, Caen, France

**Keywords:** amyotrophic lateral sclerosis, extra-motor manifestations, cognition, emotion, psychological adjustment, coping

## Abstract

It is now well recognized that, in addition to motor impairment, amyotrophic lateral sclerosis (ALS) may cause extra-motor clinical signs and symptoms. These can include the alteration of certain cognitive functions, impaired social cognition, and changes in the perception and processing of emotions. Where these extra-motor manifestations occur in ALS, they usually do so from disease onset. In about 10% of cases, the cognitive and behavioral changes meet the diagnostic criteria for frontotemporal dementia. The timecourse of behavioral and cognitive involvement in ALS is unclear. Whereas longitudinal studies have failed to show cognitive decline over time, some cross-sectional studies have demonstrated poorer cognitive performances in the advanced stages of the disease. Neuroimaging studies show that in ALS, extra-motor signs and symptoms are associated with specific brain lesions, but little is known about how they change over time. Finally, patients with ALS appear less depressed than might be expected, given the prognosis. Moreover, many patients achieve satisfactory psychosocial adjustment throughout the course of the disease, regardless of their degree of motor disability. There are scant longitudinal data on extra-motor impairment in ALS, and to our knowledge, no systematic review on this subject has yet been published. Even so, a better understanding of patients’ clinical trajectory is essential if they are to be provided with tailored care and given the best possible support. We therefore undertook to review the evidence for extra-motor changes and their time course in ALS, in both the cognitive, emotional and psychological domains, with a view to identifying mechanisms that may help these patients cope with their disease.

## Introduction

Amyotrophic lateral sclerosis (ALS) is a neurodegenerative disease of upper and lower motor neurons. In all sporadic and most genetic cases, proteinaceous aggregates of TAR-DNA binding protein 43 (TDP43) are found in these upper and lower motor neurons, as well as in certain glial cells (Saberi et al., 2015). The disease worsens relentlessly, and death occurs after a median duration of 3 years after onset and 2 years after diagnosis (Couratier et al., 2016).

All patients experience extensive and progressive muscle paralysis that results in severe functional disability, but as many as 50% may also have extra-motor signs and symptoms, including cognitive impairment (Abrahams et al., 2000; Murphy J. M. et al., 2007; Murphy J. et al., 2007) mainly affecting executive functions, emotion processing and social cognition, and behavior.

Despite being given such a gloomy prognosis, not all patients display symptoms of depression. Indeed, depression rates remain low, considering the severity of the disease (Rabkin et al., 2016), and patients’ quality of life (QoL) is not as impaired as might be expected (Roach et al., 2009; Lulé et al., 2012; Jakobsson Larsson et al., 2017). This relatively good psychosocial adjustment raises important questions about the coping strategies used by patients.

While we now have substantial knowledge about extra-motor signs of ALS at baseline, data on their clinical course remain scarce. The results of cross-sectional studies attempting to correlate disease duration and/or physical symptom severity with cognitive/psychological status suggest that, unlike motor impairment, cognitive performances are not always significantly worse in the late stages of the disease than they were in the early stages (Ringholz et al., 2005). These observations suggest that the mechanisms of psychological involvement do not simply come down to degenerative changes in the neurons of the brain. Furthermore, the fact that some studies have described patients as retaining a relatively satisfactory level of wellbeing, despite the continuous worsening of their physical condition, may seem a little surprising and deserves further consideration.

We therefore set out to review the cognitive, emotional and psychological impairments that can emerge at the beginning of the disease, and then to address the question of how they change throughout the course of the disease. We focused on ALS cases that do not meet the clinical criteria for dementia, as ALS-dementia syndromes raise different issues, and may not be as similar to ALS without dementia as is usually claimed (Lulé et al., 2018). Longitudinal studies are the most appropriate way of assessing how these impairments change, but they have several drawbacks. First, the follow-up periods are often short, and a large proportion of patients are liable to drop out, owing to the rapid progression of ALS, making it difficult to detect significant changes in extra-motor signs and symptoms over time. Second, it is difficult to find a suitable control group that accurately matches the patient sample. Third and last, the tools used to assess cognitive functioning have to be carefully chosen in order to be both adequate and usable at all stages of the disease-even the most advanced-, in order to accommodate the motor impairments that invariably interfere with testing procedures.

To fulfill the objectives of our work, we conducted a bibliographical search on PUBMED prior to the 1st October 2018 with the following keywords: Cognition, emotion, psychological adjustment, neuroimaging, longitudinal, AND ALS. We retained the articles that met our keywords, and that were written in English or in French. 190 articles were finally included in our study.

## Cognitive, Emotional, and Behavioral Changes in Als

### Cognition in ALS (Table 1)

#### Risk Factors for Developing Cognitive Deficits

Female sex, older age at onset, and low education level seem to increase the risk of cognitive impairment (Irwin et al., 2007; Montuschi et al., 2015; Flaherty et al., 2017). The presence of a *C9orf72* gene mutation is associated with more severe cognitive deficits, even in non-demented patients (Byrne et al., 2012; Montuschi et al., 2015). There are still conflicting data about the potential positive correlation between bulbar symptoms and cognitive decline, with some studies supporting this correlation (Giordana et al., 2011; Gordon et al., 2011; Strutt et al., 2012) while others don’t (Sterling et al., 2010; Zalonis et al., 2012).

**TABLE 1:** Cognition in ALS.

**Authors/Years**	**Number of patients**	**Type of study**	**Outcomes measure**	**Main findings**
Abdulla et al., 2014	58 pts / 29 NC	CS	Neuropsychological assessment Brain MRI	Global cognitive dysfunction on executive and verbal memory tests. Smaller right hippocampal volume in pts; left hippocampal volume correlates with verbal episodic memory
Abrahams et al., 2000	2 pts / 25 NC	CS	Verbal fluency Working memory	Verbal fluency impairments result from deficits in the central executive component of working memory
Abrahams et al., 2005	20 pts / 18 NC	LS : BL, 6 mo	Executive, memory, language, visuospatial functions, behavior, and emotion	Verbal fluency remains stable whereas other language abilities decreased overtime
Bock et al., 2017	49 pts	LS : BL, 7 mo	cognitive-behavioral assessment: ALS-CBS	No cognitive change whereas patients develop bi overtime
Braber, 2016	100 pts / 50 NC	LS : BL, 3 mo	Behavioral and cognitive evaluation Genetic testing	No changes over time. *C9orf72* repeat expansion has no influence on cognition
Burke et al., 2015	33 pts / 33 CG	CS	CG burden. Cognitive-behavioral profile of pts	Ci and bi (apathy and disinhibition) predict high level of CG burden
Burkhardt et al., 2017	24 pts : 21 : NC	LS : BL, 6 mo, 12-18 mo	Cognitive assessment with the ECAS and FAB	No significant alteration overtime in cognition and behavior
Byrne et al., 2012	191 pts	CS	Clinical, cognitive, behavioral, and survival data 3T high resolution MRI Screening for *C9orf72* mutation.	Mutated ALS-pts have lower age of disease onset, more often family history of FTD, more comorbid FTD, distinct pattern of non-motor cortex changes on MRI and shorter survival
Carelli et al., 2018	168 pts	CS	FAB, MoCA, ECAS BDI, STAI	Depression correlates negatively with ECAS and specially with executive functions
Consonni et al., 2013	23 pts / 39 NC	CS	Standard neuropsychological battery FBI	30%: executive ci, naming and short-term memory deficits; 20%: disorganization and mental rigidity 13%: comorbid dementia.
Dary-Auriol et al., 1997	26 pts / 26 NC	CS	Global cognition, memory, language, executive functions, MADRS	Global but subtle cognitive impairment of all neuropsychological tests with no specific profile
Elamin et al., 2011	139 pts	CS	Executive function, memory, language, visuospatial function	Executive dysfunction and comorbid FTD associated to shorter survival
Elamin et al., 2013	186: BL / 96: 2 assessments / 46: 3 assessments	LS :	Cognitive assessment	Cognitive function declines faster in patients cognitively impaired at BL.
Flaherty et al., 2017	161 pts	CS	Cognitive-behavioral profile × site of onset and gender relative to emerging FTD	Bulbar pts : worse letter fluency; Bulbar females : worse category fluency; females with low oestrogen levels: worse letter fluency
Gillingham et al., 2017	20 pts / 36 NC	CS	ALS-CFB	Executive dysfunction
Gordon et al., 2011	131 pts	Cross-sectional	Spectrum and clinical associations of ci impairment in ALS Effect of ci on survival	40% ci, 10%FTD Impaired patients: less education, more likely to have bulbar onset. Severe cognitive impairment predicts shorter survival
Govaarts et al., 2016	110 pts	CS	Behavioral and cognitive evaluation	Frontal syndrome correlates negatively to survival
Hervieu-Bègue et al., 2016	15 pts	CS	Semantic memory	60% of pts have semantic memory impairment
Hu et al., 2013	37 pts / 33 NC	CS	ALS-BCA and other neuropsychological tests	Shorter survival associated to dementia and behavioral impairment
Iwasaki et al., 1990	22 pts / 18 NNMC / 17 NC	CS	MMSE; immediate and delayed memory tests	Pts perform lower than NNMC and NC at MMSE and memory tasks. MMSE and memory correlates negatively to upper limb function
Kamminga et al., 2016	20 ALS pts / 15 ALS-FTD / 27 PNFA / 23 NC	CS	Syntax comprehension : Test for Reception of Grammar Brain volume by MRI-VBM	Syntactic comprehension impaired in 25% of ALS, 92.9% of FTD-ALS, and 81.5% of PNFA Impairment correlates with left peri-insular atrophy
Kasper et al., 2015	98 pts / 70 NC	CS	Executive cognitive, Executive behavior	70% of ci pts have executive dysfunction (initiation and shifting). Dominant bi is apathy
Kasper et al., 2016	93 pts : 73 NC	LS : BL and at three time every 3-6 mo	Executive functioning	No significant decline
Leslie et al., 2015	17 pts / 19 ALS-FTD, 22 SD / 26 NC	CS	Assessment of semantic deficits Brain volume by MRI-VBM	Significant semantic deficits in ALS and ALS-FTD compared to controls. Severity of semantic deficits varies across clinical phenotypes. Anterior temporal lobe atrophy correlates with semantic deficits
Kilani et al., 2004	18 pts : 19 NC	LS : BL, 6 and 12 mo	Cognitive function	Executive alteration at baseline does not worsen at follow-up
Machts et al., 2018	31 pts and 29 NC	CS	Brain MRI	Reduction of left and right hippocampal volumes in patients’ *cornu ammonis* field 1 (CA1)
Montuschi et al., 2015	207 pts / 127 NC	CS	Comprehensive neuropsychological assessment	49.7% cognitively normal, 12.6% ALS with FTD, 19.7% ALS-executive ci, 5.5% ALS-non executive ci, 16% ALS-bi and 6% non-classifiable ci. ALS-FTD older, lower educational level and shorter survival
Murphy J. M. et al., 2007	23 pts	CS	Neuropsychological, neurobehavioral assessment	No impairment: 11 pts; behavioral changes: 4; FTD: 5;other :3 (Alzheimer : 1)
Murphy et al., 2016	274 pts	CS	Neuropsychological and neurobehavioral assessment : ALS-CBS	54.2% ci, 14.1% bi and 6.5% FTD
Palmieri et al., 2015	260 pts / 134 NC	CS	Executive function, memory and language.	29% pts have executive ci and 18% non-executive ci; Females have 2-fold risk to have executive ci
Olney et al., 2005	81 pts	CS	Survival predictors	Younger age, limb onset and absence of comorbid FTD predicted higher survival
Phukan et al., 2012	160 pts / 110 NC	CS	Comprehensive neuropsychological battery	46% no ci; 14% FTD; 21% executive ci; 14% non-executive ci
Poletti et al., 2018	164 BL / 48 at 6 mo / 18 at 12 mo / 5 at 18 mo	LS : BL, 6 mo, 12 mo, 24 mo	Cognitive and behavioral examination : ECAS	No behavioral or cognitive worsening
Raaphorst et al., 2015	26 pts / 21 NC	CS	Neuropsychological assessment Brain MRI	Prose memory impairment correlates to hippocampal volume
Rabkin et al., 2016	247 pts	CS	Cognition-behavior : CBS Psychological : PHQ	40 % ci, 9% bi, 18% ci and bi, 12 % Major or minor depression; 12% Bi associated with depression
Ringholz et al., 2005	279 pts / 129 NC	CS	Neuropsychological testing	49% intact; 32% mild ci, 13% moderate ci, 6% severe ci, 15% FTD
Roberts-South et al., 2012	16 pts / 12 NC	LS : BL, 6-12-18-24 mo	Language testing with standardized tests and analysis of productivity and content	No alteration at standardized tests. Impairment of discourse content. Alteration of performances overtime
Robinson et al., 2006	19 pts / 8 CG	LS : BL, 6 mo	Neuropsychological assessment	No change overtime even if some patients develop abnormalities
Schreiber et al., 2005	52 pts	LS : BL and each 4 months until 18 mo	Executive functions, memory and attentional control.	No decline on follow-up
Stojkovic et al., 2016	58 pts	LS : BL and yearly	Executive function and correlation to survival	49.5 % executive ci and BL executive status might predict survival
Strong et al., 1999	13 pts	CS	Neuropsychological, language and speech testing	Mild impairment in several domains especially when bulbar onset
Strutt et al., 2012	44 pts	CS	Comprehensive pulmonary (vital capacity) and neuropsychological assessments	More respiratory-impairment when clinically significant impairments in frontal-lobe-mediated behaviors. Greater executive functioning deficits in patients with bulbar versus limb onset
Xu et al., 2017	108 pts / 60 NMC	CS	ACE-III, FAB, ECAS, ALS-FTD-Q, MiND-B	14 to 30% ci on ALS and 3.3 to 11.7 % on NMC. 32 % bi on ALS and 39 % on NMC. Ci and bi influence prognosis
Wei et al., 2016	91 pts	CS	Neuropsychiatric symptoms and cognition: NPI, ACE-R, FAB	Depression 59%, anxiety 41%, lability 26%. NPI correlates with ACE-R but not with FAB
Woolley et al., 2018	294 BL / 134 at follow up	LS : BL, 5-18 mo	Cognitive and behavioral changes	Worsening of behavior but not cognition
Zalonis et al., 2012	48 pts / 47 NC	CS	Executive function: TMT, SNST, WAIS, WCST	Pts worse than NC on TMT, SNST and WAIS Similarities. No difference between bulbar and spinal onset pts

*ALS, Amyotrophic Lateral Sclerosis; ACE, Addenbrooke’s Cognitive Examination; ALS-BCA, ALS Brief Cognitive Assessment; ALS-CBS, ALS Cognitive Behavioral Screen; ALS-CFB, ALS-Computerized Frontal Battery; ALS-FTD, ALS-Frontotemporal Dementia; ALS-FTD-Q, ALS-Frontotemporal Dementia Questionnaire; BDI, Beck’s Depression Inventory; Bi, Behavioral Impairment; BL, Baseline; Ci, Cognitive Impairment; CG, Caregiver; Cibi, Cognitive and Behavioral Impairment; CS, Cross-Sectional Study; ECAS, Edinburgh Cognitive and Behavioral ALS Screen; FAB, Frontal Assessment Battery; FTD, Frontotemporal Dementia; LS, Longitudinal Study; MADRS, Montgomery-Asberg Depression Rating Scale; MiND-B, Motor Neuron Disease Behavioral instrument; MMSE, Mini Mental State Examination; MRI, Magnetic Resonance Imaging; NC, Normal Controls; NMC, Controls With Neuromuscular Disease, NNMC, Non Neurological Medical Controls; NPI, Neuropsychiatric Inventory; PHQ, Patient Health Questionnaire; PNFA, Progressive Nonfluent Aphasia; Pt, Patient; SD, Semantic Dementia; STAI, State-Trait Anxiety Inventory; SNST, Stroop Neuropsychological Screening Test; TMT, Trail Making Test; VBM, Voxel-Based Morphometry; WAIS, Similarities Subtest of The Wechsler Adult Intelligence Scale.*

The presence of depressive symptoms also seems to exacerbate the executive deficit in patients, with a negative correlation between scores on depression scales and those on cognitive tests (Wei et al., 2016; Carelli et al., 2018). These findings are not surprising, given the negative impact that depression is known to have on cognitive functions, in terms of attention and memory (Bortolato et al., 2016).

#### Baseline

##### Frequency and profile

Cognitive impairment occurs in 30–50% of patients with ALS, depending on the study and the neuropsychological tools used to assess cognitive functions (Giordana et al., 2011; Zago et al., 2011; Byrne et al., 2012; Kasper et al., 2015; Beeldman et al., 2016; Murphy et al., 2016). The cognitive deficit profile includes impairment of executive functions, verbal fluency, language, social cognition and verbal memory (Phukan et al., 2012; Beeldman et al., 2016). In 6–14% of patients, the cognitive impairment meets the criteria for a behavioral variant of frontotemporal dementia (FTD) (Consonni et al., 2013; Montuschi et al., 2015; Murphy et al., 2016). In the study by Ringholz et al. (2005), 51% of patients were cognitively impaired, compared with 5% of controls, and 14% met the diagnostic criteria for FTD. A cluster analysis indicated four patient subgroups: 49% with intact cognition, 32% with mild cognitive impairment, 13% with moderate impairment, and 6% with severe impairment.

Executive alterations affect verbal fluency, attention monitoring, switching, working memory, cognitive flexibility and mental control, and reasoning and coordinating rules (Phukan et al., 2012; Beeldman et al., 2016). Other executive functions are also impaired, such as initiation and shifting (Kasper et al., 2015). A study by Palmieri et al. (2015) found that female patients with ALS were twice as likely as males to have dysexecutive dysfunction-an intriguing finding that is not clearly explained to date.

Cognitive alterations observed in ALS also include language impairments (Iwasaki et al., 1990; Raaphorst et al., 2010; Giordana et al., 2011). According to Woolley and Rush (2017), language may be altered in 30–40% of patients without dementia, regardless of executive dysfunction, dysarthria or respiratory failure. Linguistic impairments may include deficits in syntactic processing, verb naming and action verb processing, semantic and verbal paraphasias, and syntactic comprehension deficits. Kamminga et al. (2016) found that syntactic comprehension was defective in 25% of patients with ALS without dementia. Leslie et al. (2015) observed semantic deficits in 35% of not demented ALS patients, and Hervieu-Bègue et al. (2016) in as many as 60% of such patients. Woolley and Rush (2017) suggested that language alterations observed in ALS reflect the fact that ALS and nonfluent/agrammatic primary progressive aphasia lie on a pathogenesis continuum.

Memory may also be altered to some extent in ALS (Dary-Auriol et al., 1997; Abrahams et al., 2000; Neary et al., 2000; Ringholz et al., 2005), but the nature of this impairment is subject to debate. It has been suggested that defective memory is mainly the consequence of executive dysfunction, especially since memory deficits very rarely occur in isolation in ALS (Strong, 2017). There is, however, some evidence in favor of hippocampal involvement. Abdulla et al. (2014) found reduced hippocampal volume in a series of patients with ALS relative to controls, and Machts et al. (2018) showed that this reduction mostly affects the anterior part, including the CA1 field-a critical structure for episodic memory. Furthermore, Raaphorst et al. (2015) found that immediate and delayed story recall scores were below normal in 23% of patients with ALS, and these performances were correlated with hippocampal gray-matter volume. Further studies are needed to try to determine the real status of memory in ALS.

The Strong (2017) revised diagnostic criteria of ALS-FTSD classify ALS into several categories according to the type and severity of neuropsychological impairments: pure ALS (no impairment), ALSci (cognitive impairment), ALSbi (behavioral impairment), ALScibi (cognitive and behavioral impairment), all three without dementia, and ALS-FTD. According to those criteria, the diagnosis of ALS-ci requires either executive dysfunction or language impairment or both, and the diagnosis of ALS-bi requires either apathy or two of the Rascovsky criteria for FTD.

#### Over Time

Most longitudinal studies of cognitive changes in patients with ALS have a follow-up period of 6 months. A number of authors have reported an absence of decline in cognitive functions during the course of the disease (Kilani et al., 2004; Schreiber et al., 2005; Braber, 2016; Kasper et al., 2016; Burkhardt et al., 2017; Poletti et al., 2018; Woolley et al., 2018). Although patients may initially have lower performances than controls, their deficits remain stable over time. It has been argued, however, that unlike normal controls, patients with ALS do not display a *practice effect* in repeated assessments with the Edinburgh Cognitive and Behavioural ALS Screen (ECAS) battery, which has been interpreted as evidence of a *pre-symptomatic* cognitive decline (Burkhardt et al., 2017). Abrahams et al. (2005) found that while most cognitive scores remained stable over time (including written and spoken verbal fluency) patients with ALS performed a single word retrieval test increasingly slowly whereas controls performed it faster. Furthermore, the caregivers of patients report increasing cognitive dysfunction in daily life over time, unlike controls’ partners. Robinson et al. (2006) found that cognitive changes occurred in 36% of patients with ALS over a 6-month period. Cognitive status seems to have a heterogeneous outcome in ALS. Behavioral symptoms may appear in patients with stable cognitive performances (Bock et al., 2017). The presence of even mild cognitive or behavioral impairment (as defined by the 2017 Strong criteria) at baseline seems to be a significant risk factor for the later appearance of a full-blown frontotemporal syndrome (Elamin et al., 2013). The choice of the neuropsychological tests used to assess cognitive status is of primary importance, as Roberts-South et al. (2012) suggested in their longitudinal study of discourse changes in a group of patients with ALS compared with healthy participants over 24 months. Subtle cognitive language deficits affecting discourse (content rather than productivity) were found to emerge early in ALS and worsen as the disease progressed. The authors concluded that language deficits are more thoroughly detected by the discourse analysis method than by standard language tasks. Gillingham et al. (2017) assessed patients with ALS at baseline and 9 months later using both standardized non-specific cognitive tests and the ALS-Computerized Frontal Battery (ALS-CFB), which was specifically designed for patients with ALS. While the basic cognitive tests failed to reveal any change over time, the ALS-CFB showed a significant decrease in cognitive performances in patients compared with controls (e.g., for verbal fluency). Finally, patients with bulbar onset seem to exhibit a progressive decline in cognitive functions (Strong et al., 1999).

It is therefore difficult to give a simple answer to the question “do cognitive functions decline over time in ALS?” owing to the heterogeneity of the patients and the difficulty of differentiating genuine cognitive deficits from the consequences of the steadily worsening motor impairment. A tentative, preliminary answer could be that when ALS is associated with a cognitive impairment at baseline, this impairment is likely to progress, and when dementia is present at diagnosis, decline is faster (Woolley and Katz, 2008). Normal cognition at baseline was associated with tendency to remain cognitively intact overtime (Elamin et al., 2013).

#### Consequences for Patients and Caregivers

Cognitive impairment in ALS is associated with a more rapid progression of the disease and a poorer prognosis, with reduced survival (Elamin et al., 2011; Giordana et al., 2011; Gordon et al., 2011; Hu et al., 2013; Xu et al., 2017). Stojkovic et al. (2016) found that the death risk was increased threefold by the presence of executive dysfunction in ALS. Poorer survival could be explained by patients’ difficulty weighing up the benefits of non-invasive ventilation (Govaarts et al., 2016), or their reduced compliance in the use of medical devices (Olney et al., 2005). The correlation between the severity of bulbar symptoms and the cognitive deficit may also influence survival figures (Olney et al., 2005). Elamin et al. (2013) evaluated the clinical impact of cognitive deficit in patients with ALS in a longitudinal study. Executive impairment at the initial consultation was associated with significantly higher rates of attrition due to disability or death, and faster rates of motor functional decline, particularly bulbar function. QoL in patients has been found to be worse in the case of cognitive impairment (Hu et al., 2013). Caregivers are the pillars of patient care. They may be the spouse, children, brothers or sisters. It is cognitive and behavioral impairment, rather than the patients’ physical disability, that increases caregivers’ burden and anxiety (Rabkin et al., 2009; Burke et al., 2015). This underscores the importance of screening patients with ALS for cognitive dysfunction, in order to predict disease progression and provide more adequate care.

#### Neuroimaging Correlates of Cognitive Deficits

Anatomical brain changes are more pronounced in patients with ALS who exhibit cognitive impairment than in those who do not. Gray matter volume in the frontal and temporal lobes is reduced when cognitive impairment is present (Abe et al., 1997; Giordana et al., 2011; Mioshi et al., 2013), as is that of the cerebellar cortex and basal ganglia (Christidi et al., 2018). Reduced cortical thickness is observed in the bilateral precentral gyrus, insular and cingulate cortices, and frontotemporal regions in the case of cognitive deficit (Schuster et al., 2014a; Agosta et al., 2016). Left peri-insular atrophy was found to correlate with scores on a syntactic comprehension task (Kamminga et al., 2016). There are greater white-matter changes in the corticospinal and corpus callosum tracts when cognitive functions are altered (Sarro et al., 2011), and these extend to extra-motor tracts, particularly within the frontal lobes and associative areas including the cingulum and the inferior longitudinal, inferior fronto-occipital, and uncinate fasciculi (Sarro et al., 2011; Kasper et al., 2014; Christidi et al., 2018). Verbal learning and memory test scores are correlated with white-matter values in the fornix (Sarro et al., 2011). Cerebral regional metabolism has been repeatedly studied with fluorodeoxyglucose positron emission tomography (18FDG-PET) in patients both with and without cognitive dysfunction. One of the most recent studies shows that cognitively impaired (but not demented) patients with ALS have relative hypometabolism in the right cingulate and frontal cortex, and bilaterally in the prefrontal cortex, compared with patients with no cognitive impairment (Canosa et al., 2016). These patients also have relative hypermetabolism in parts of the midbrain and corticospinal tracts (Canosa et al., 2016). In a combined MRI and PET study in patients with ALS, Buhour et al. (2017) found gray-matter atrophy, predominantly in the temporal poles, and hypometabolism in the left superior medial cortex. Hypermetabolism was also found in parts of the temporal lobes and the cerebellum. A series of negative correlations between cognitive performance and regional cerebral metabolism in functionally relevant areas suggest that hypermetabolism is more likely to reflect deleterious processes such as neuro-inflammation rather than compensatory neuronal activity.

Longitudinal studies of anatomical cerebral changes in ALS with cognitive modifications remain rare, for the same reasons as those dealing with purely clinical aspects of the disease. Floeter et al. (2016) assessed brain volume changes in patients who were either sporadic or carriers of the genetic mutation C9orf72 (C9+), which is often associated with FTD or cognitive impairment. These authors found that over a 6-month period, ventricular volume increased in C9+ versus sporadic cases, suggesting that subcortical involvement influences cognitive performances in this particular group of patients. Other microstructural changes over time in ALS have been documented. A significant decline in the cortical thickness of frontal, temporal and parietal regions is observed over time, whereas the reduced cortical thickness of the precentral gyrus at the beginning of the disease remains stable (Verstraete et al., 2012; Schuster et al., 2014b). Menke et al. (2018) assessed brain changes in patients with ALS over 2 years and reported widespread changes in both white and gray matter in the cingulate gyrus, thalami, caudate nuclei, pallidum, hippocampi and parahippocampal gyri, and insula. These results indicate that over time, cerebral changes extend into extra-motor areas, but it remains difficult to draw a link between these changes and clinical cognitive implications.

### Changes in Emotion Perception and Social Cognition (Table 2)

#### Baseline

##### Perception of emotions

A number of studies have shown that emotion perception is impaired in ALS (Lulé et al., 2005; Zimmerman et al., 2007; Palmieri et al., 2010; Girardi et al., 2011), with patients exhibiting deficits in emotion recognition (facial or prosodic) and emotional valence attribution, and decreased excitability when emotional material is presented. By contrast, Cavallo et al. (2011) and Papps et al. (2005) found no deterioration in either facial emotional recognition or judgments of emotional valence. Clinical features such as type of onset and disease severity may explain the heterogeneity of patients’ emotional deficit profiles, as suggested by Sedda (2014). Oh et al. (2016) confirmed the presence of facial emotion recognition deficits in ALS. Bora (2017) carried out a meta-analysis of 15 studies of emotion recognition in ALS and concluded that ALS is associated with significant impairments in facial emotion recognition, especially for disgust and surprise.

**TABLE 2:** Social cognition and emotion perception in ALS.

**Authors/year**	**Pts/NC**		**Outcome measures**	**Main findings**
Andrews et al., 2017b	33 pts / 22 NC	CS	Emotion processing multimodal tasks (facial affect and voice prosody) Executive, mood and functional tests	Difficulties in recognizing emotions both in faces and voices
Benbrika et al., 2018	28 pts / 30 NC	CS	20-item Toronto Alexithymia Scale Correlation / gray matter volume	Pts > NC. Alexithymia correlated with prefrontal cortex, right temporal pole and parahippocampal gyri
Burke et al., 2016	106 pts/50 NC	CS	RME and executive function in bulbar vs. spinal-onset ALS	Bulbar onset pts have more social cognition but not more executive impairment than spinal onset pts.
Carluer et al., 2015	23 pts / 23 NC	CS	An original false-belief task and executive tasks 18F-FDG PET-scan examination	ToM impairment only partially linked to executive dysfunction. Correlated with metabolism of dorso-medial and dorsolateral prefrontal cortices, and SMA
Cavallo et al., 2011	15 pts / 21 NC	CS	Social cognition (private vs. social intentions)	Impaired comprehension of social context
Gibbons et al., 2007	16 pts / 16 NC	CS	ToM and executive function	Abnormalities of social cognition linked to executive function
Gillingham et al., 2017	20 pts / 36 NC at baseline 11 pts / 20 NC after 9 months	LS	ALS- Computerised Frontal Battery	Impairment in social cognition, initiation of behavior, executive processing and response suppression. Decline in executive processing over time
Girardi et al., 2011	19 pts/20 NC	CS	Behavior (FSBS) Social cognition (modified IGT) Gaze, RME, emotion recognition	Increased apathy. Different profile from NC. Impaired emotion recognition
Lulé et al., 2005	12 pts / 18 NC	CS	Judgment of pictures from the IAPS	Pts more positive than NC
Meier et al., 2010	18 pts / 18 NC	CS	Orbitomedial prefrontal tasks (Faux Pas, emotional prosody recognition, reversal of behavior in response to changes in reward, decision making and Neuropsychiatric Inventory Dorsolateral prefrontal tasks (verbal and written fluency and planning)	Dissociations involving either one or two or both of the orbito-frontal or dorsolateral prefrontal regions. Variability and heterogeneity of cognitive involvement in ALS
Oh et al., 2016	24 pts / 24 NC	CS	K-MMSE,BDI, FAB Perception of emotional expression	Pts < NC
Palmieri et al., 2010	9 pts / 10 NC	CS	Two fMRI emotional attribution and memory tasks	Activation increased in the left hemisphere and reduced in the right one in both tasks
Papps et al., 2005	19 pts / 20 NC	CS	Facial expression recognition, Social judgement rating of faces, Memory for emotional words.	No enhanced recognition memory for emotional vs. neutral words
Roy-Bellina et al., 2008	14 pts / 9 NC		20-item Toronto Alexithymia Scale	Pts > NC
Trojsi et al., 2016	22 pts / 15 NC	CS	ToM: Emotion Attribution Task, Advanced Test of ToM, Eyes Task Executive, verbal comprehension, visuospatial tasks, behavior, and QoL	Impairment of both affective and cognitive ToM that impacts the “Mental Health” component of QoL
Trojsi et al., 2017	21 pts / 15 NC at baseline and after 6 months	LS	Affective and cognitive ToM and global neuropsychological assessment, Resting state MRI study	No impairment at baseline. At 6 months, impairment of both affective and cognitive ToM in bulbar onset pts, and of the cognitive subcomponent alone in spinal onset pts. Various changes in RSN connectivity.
van der Hulst et al., 2015	33 pts / 26 NC	CS	Social cognition (Cognitive–Affective Judgement of Preference Test), Measures of empathy and awareness	Affective and ToM deficit, poor empathy and self-awareness of their difficulties
Watermeyer et al., 2015	55 pts / 49 NC	CS	Social cognition, executive functions, mood, behavior and personality	Social cognition impairment mainly predicted by executive dysfunction
Zimmerman et al., 2007	13 bulbar pts / 12 NC	CS	Facial emotional and prosodic recognition task	Pts < NC

*IGT, Iowa Gambling Task; fMRI, Functional MRI; FDG, Fluorodeoxyglucose; PET, Positron Emission Tomography; ToM, Theory of Mind; FSBS, Frontal Systems Behavior Scale; RME, Reading the Mind in The Eyes; K-MMSE, Korean Version of the Mini Mental State Examination; QoL, Quality of Life; RSN, Resting-State Network; SMA, Supplementary Motor Area. For more please refer to Table 1.*

Patients’ processing of their own emotions (alexithymia) also seems to be altered, although there have been very few studies so far in this area. Roy-Bellina et al. (2008) reported that patients with ALS are more often alexithymic than controls. Benbrika et al. (2018) assessed a group of patients with ALS and a matched control group on the three dimensions of alexithymia: Difficulty Identifying Feelings, Difficulty Describing Feelings, and Externally-Oriented Thinking. Patients were more often alexithymic than controls and had a higher level of alexithymia, especially on the Difficulty Identifying Feelings dimension, suggesting that they have difficulty with the first stage of own emotion processing, namely recognizing one’s own emotions.

##### Social cognition

Social cognition is a set of cognitive processes used to encode, decode, store, retrieve and use information about people in social relationships. It has several dimensions, such as theory of mind (ToM), empathy, and moral reasoning. ToM refers to the ability to infer the mental and emotional states (i.e., beliefs, preferences and intentions) of oneself and others, and contributes to the understanding of other people’s behavior. It can be divided into cognitive and affective ToM. Studies of ToM abilities in ALS have sometimes yielded conflicting results, owing to the heterogeneity of the tasks used and the patients’ cognitive status, or the presence of depressive symptoms.

Early studies in this domain attested to a deficit in ToM, showing that patients with ALS perform more poorly than healthy individuals on the faux pas task (task assessing cognitive ToM through stories featuring appropriate and inappropriate social behavior), story comprehension task (strip stories presented to participants who have to choose the most appropriate picture) and decision making task (task evaluating decision making abilities under ambiguity in a card game) (Gibbons et al., 2007; Meier et al., 2010; Cavallo et al., 2011; Girardi et al., 2011).

More recently, van der Hulst et al. (2015) investigated whether the ToM deficit described in ALS could be further delineated as one of either affective or cognitive ToM, and explored the relationship between this social cognition deficit and the behavioral manifestations of empathy and self-awareness. Patients were evaluated on a neuropsychological battery that included a cognitive–affective judgment of preference test, a questionnaire to evaluate their self-awareness, and the Neuropsychiatric Inventory–Questionnaire (NPI-Q) to pick up any behavioral changes. The authors found deficits in both cognitive and affective ToM (affective + cognitive ToM deficit in 36% of patients, affective ToM deficit in 12%, and cognitive ToM deficit in 3%). Patients with a ToM deficit were more likely to display behavioral changes such as apathy, lack of empathy, and low self-awareness. When Carluer et al. (2015) compared 23 non-demented patients with controls matched for age, sex and educational level on cognitive ToM, by administering a false-belief task, they found a cognitive ToM deficit in patients with impaired executive functions. Trojsi et al. (2016) assessed ToM in patients with ALS using a comprehensive battery of specific tasks: the Advanced Task of ToM (ATM), in which participants listen to stories and then have to explain the protagonists’ actions; the Emotional Attribution Task (EAT), in which participants have to identify the emotions experienced by the protagonists in a story; and the Reading the Mind in the Eyes task (Eyes test or RME). Patients also underwent a cognitive battery and a QoL assessment. Patients scored lower than controls on the EAT but not on the ATM, and EAT and RME correlated positively with the education, prose memory and mental health items of the QoL questionnaire. Patients were not very cognitively impaired. By contrast, Andrews et al. (2017b) reported impaired complex facial and prosodic emotion recognition, but intact simple facial affect recognition, in a group of non-demented patients with ALS. Finally, Woolley and Rush (2017) showed that the performances of patients without dementia may be impaired on tasks of complex facial affect recognition or affective prosody recognition, and may have difficulty interpreting the gaze direction of others.

To summarize findings on social cognition in ALS, even though results are quite divergent, affective ToM seems to be systematically affected, whereas scores on cognitive ToM seem to be underpinned by patients’ cognitive profile. Studies including patients with severe cognitive impairment have reported cognitive ToM deficits (Carluer et al., 2015), whereas those among patients with only mild or moderate cognitive impairment point to preserved cognitive ToM (Trojsi et al., 2016). This prompts the question of a link between social cognition and other cognitive processes.

##### Interactions between cognitive status, social cognition, and emotion recognition

Most studies investigating the pattern of interaction between social cognition impairment and other cognitive functions support the idea of an interaction between executive functions and cognitive ToM, with positive correlations between executive functions (verbal fluency, composite executive score, executive score of the Frontal System Behavior Scale, etc.) and ToM (decision making task, faux pas task, etc.) (Gibbons et al., 2007; Meier et al., 2010; Watermeyer et al., 2015). In Carluer et al. (2015) study, cognitive ToM scores were only partially associated with executive performances, notably shifting, inhibition, and the ability to manipulate items in working memory. This led the authors to propose that the ToM deficit observed in some cases of ALS is not simply the consequence of an executive function impairment. Nevertheless, as studies do not generally exclude patients who have only a mild cognitive impairment, it is still unclear whether any ToM deficit is associated with executive deficit in ALS or whether ToM *per se* is disturbed.

Results on the interaction between emotion recognition and affective ToM performances and cognitive abilities have been somewhat conflicting. In a subset of patients with bulbar ALS, Zimmerman et al. (2007), found that 62% of them had emotion recognition defects, with no correlation with cognitive symptoms. However, the cognitive assessment was done using the Mini-Mental State Examination (MMSE), which is not well suited to patients with ALS. When they calculated a composite executive score, Watermeyer et al. (2015) also found no correlation between cognitive performances and RME test scores. By contrast, Girardi et al. (2011) reported a negative correlation between RME performances and *z* scores for verbal fluency. Burke et al. (2016) compared the RME test performances of three groups of patients: “no cognitive impairment,” “defect in only one cognitive domain,” and “multi-executive deficits/cognitively impaired.” The patients with no cognitive impairment performed better than those with a defect in only one cognitive domain, who in turn performed better than those in the third group. Finally, Andrews et al. (2017b) found that crossmodal (facial and prosodic integration) emotion recognition correlated with executive functions, whereas the separate modalities of emotion recognition did not. No specific cognitive function seems to be related to either affective ToM or emotion recognition, and as Trojsi et al. (2016) study suggests, the link between ToM performances and cognitive impairment may only concern the cognitive aspect of ToM.

#### Over Time

To our knowledge, only two studies have addressed changes in emotional and ToM abilities and their neural correlates in the course of ALS. Gillingham et al. (2017) assessed cognitive and emotional changes in patients with ALS at baseline and 9 months later using a specific cognitive and emotional battery (ALS-CFB) and other standardized cognitive tests, including cognitive first-order and cognitive and affective second-order ToM tasks. At baseline, patients showed a deficit in emotion perception for happy emotions, and scored significantly lower than controls on the first-order cognitive ToM task. At 9 months, in the emotion perception task, patients were better at recognizing angry faces than controls were, but there were no changes over time in ToM. Trojsi et al. (2017) assessed cognitive and affective ToM at baseline and 6 months later. At baseline, no ToM abnormalities were found in patients, whereas at follow up, patients with bulbar onset exhibited a decline in both affective and cognitive ToM, whereas those with limb onset only displayed impairment of cognitive ToM over time. This study also included an fMRI investigation, only at baseline, that will be discussed in the next section about neuroimaging. This result is in line with the more extensive prefrontal hypometabolism observed in patients with bulbar versus limb onset by Cistaro et al. (2012).

#### Neuroimaging Correlates of Emotional and Social Cognition Changes

Emotion recognition impairment is associated with alteration of white-matter integrity along the right inferior longitudinal fasciculus and inferior fronto-occipital fasciculus (Crespi et al., 2014). Defective emotional empathy attribution is correlated with reduced gray-matter density in the anterior cingulate cortex and right inferior frontal gyrus (Cerami et al., 2014). Palmieri et al. (2010) found a general increase in left-hemisphere activation and reduced right-hemisphere activation in patients when they were asked to attribute an emotional valence or remember an emotion. Aho-Özhan et al. (2016) assessed emotion recognition and its functional neural correlates in a group of patients with ALS and a matched control group. Patients recognized disgust and fear less accurately, and had lower activity in the hippocampus on both sides (brain regions involved in negative emotion processing) and more activity in the right inferior frontal gyrus.

Using a composite executive function score as a covariate, Carluer et al. (2015) found positive correlations between cognitive ToM performances and the metabolism of the bilateral superior frontal gyrus, bilateral middle frontal gyrus, and bilateral supplementary motor area. Buhour et al. (2017) sought to understand the metabolic dysfunction and its neurobehavioral consequences better by subjecting a sample of 37 patients with ALS to a comprehensive neuropsychological assessment and PET imaging. Significant negative correlations were found between metabolic activity within the left fusiform gyrus and performance on a false-belief task.

Longitudinal studies are very few and far between. Lulé et al. (2007) assessed emotional valence attribution, arousal, association of movement, and brain functioning when emotional material was presented to patients with ALS and healthy controls at baseline and 6 months later. Patients had an increased brain response in the right supramarginal area and a reduced brain response in extrastriatal visual areas at both measurement points, compared with healthy controls. In the patient group, a reduced brain response in the anterior insula at follow up was correlated with subjective arousal. This reduced response was tentatively interpreted as indicating reduced arousal during the course of the disease at the neural and behavioral levels. The reduced activity in extrastriatal visual areas could be similarly interpreted. The increased brain response in the right supramarginal area could represent altered sensitivity to social-emotional cues.

In the aforementioned study of ToM, Trojsi et al. (2017) assessed, only at baseline, the resting state functional connectivity with fMRI in 21 ALS patients compared to a matched group control. Subjects also underwent affective and cognitive ToM tasks both at baseline and after 6 months. Compared to controls, ALS patients exhibited abnormalities (1) within the DMN (Default mode network) with decreased connectivity in anterior node and increased connectivity in posterior node, (2) within the right FPN (Fronto-parietal network) with decreased connectivity in the supramarginal gyri and (3) within the left FPN and SLN (salient network) with decreased connectivity on the medial and dorsolateral prefrontal cortices. Positive correlations were found between affective ToM performances and functional connectivity of the posterior node of the DMN and the supramarginal gyri. ToM alterations were associated to decreased connectivity in the posterior cingulate cortex (PCC) and the occipital gyri of DMN. The authors suggest that these results support the hypothesis of the potential role of frontotemporoparietal network structures, such as the PCC and the supramarginal gyri in reasoning about the contents of another person’s mind particularly in affective mentalizing and in empathetic face processing.

### Behavioral Changes (Table 3)

#### Baseline

##### Frequency and typology

Behavioral changes are increasingly being recognized as a common feature in ALS, and may be similar to those observed in FTD. They occur in 24–69% of patients with ALS (Murphy J. M. et al., 2007; Gibbons et al., 2008; Witgert et al., 2010; Lillo et al., 2011; Bock et al., 2016; Burke et al., 2017), 6–25% of whom meet the criteria for FTD (Murphy J. et al., 2007; Lillo et al., 2011; Bock et al., 2016; Burke et al., 2017). In some patients, they may appear as early features, even prior to the development of the motor symptoms (Mioshi et al., 2014).

**TABLE 3:** Behavioral changes in ALS.

**Authors/year**	**N**	**Type**	**Outcomes measures**	**Main findings**
Andrews et al., 2017a	40 pts / 40CG and 27 NC & relatives	CS	Behavioral changes: CBI-R CG burden	Pts: disturbance on everyday skills, self-care, and sleep, mood and motivation. CG burden: pts’ skills, motivation and memory
Bock et al., 2016	86 pts	CS	Cognitive-Behavioral Screen Patient QoL CG burden Disease stage	Cognitive impairment: 62%; Behavioral impairment: 37%; FTD: 5% Severity of deficits not associated with patient QoL; predicts higher CG burden. Self-reported QoL lower in pts with depressive symptoms and more advanced disease
Bock et al., 2017	49 pts BL & 7 mo	LS	Assessment of cognitive-behavioral function using the ALS-CBS	Cognitive status: no change over time; Pts initially classified as behaviorally normal show increased behavioral problems over time
Burke et al., 2017	317 pts / 66 NC	CS	Behavioral changes: BBI Cognitive assessment, Impact on survival	Behavioral changes: none, 57%; mild to moderate: 30%; severe (FTD) : 13% Behavioral changes predicted by social cognitive performances. No impact on survival
Caga et al., 2018	60 pts	Cs	Impact of apathy (AES) on QoL (PWI)	Apathy: 30 %. Pts with apathy have poorer overall QoL
Consonni et al., 2013	23 pts, 11 Lower MND, 39 NC	CS	Cognitive and behavioral assessment	Executive dysfunction: 30% of ALS pts disorganization and mental rigidity: 20% Dementia:13%. No correlation between cognitive and behavioral changes and clinical features
Crockford et al., 2018	161 pts, 80 NC	CS	Pts: ECAS; Disease stage : the King’s Clinical Staging System CG behavioral interview	Behavioral impairment : 40% (firstly apathy). Higher number of behavioral features found across advancing stages.
Femiano et al., 2018	22 pts / 19 NC	CS	Apathy: AES, FrSBe, Global cognitive assessment, Brain imaging: DTI	No behavioral and cognitive impairment. Apathy inversely correlated to fractional anisotropy (FA) in several WM areas
Floeter et al., 2017	34 pts at BL, 6, 12, 18 mo	LS	Cognitive and behavioral manifestation in carriers of the mutation *C9orf72*: letter fluency and FBI-ALS	Symptomatic carriers decline at each evaluation on cognitive and behavioral functioning
Gibbons et al., 2008	16 pts	CS	The Manchester FTD Behavioral Interview of informants	Behavioral changes: 87%; FTD:8% Behavioral changes associated to bulbar palsy, but not to disease duration
Kasper et al., 2015	98 pts / 70 NC	CS	Executive cognitive, Executive behavior	70% of ci pts have executive dysfunction (initiation and shifting). Dominant bi is apathy
Grossman et al., 2007	45 pts	CS	FrSBe, Verbal fluency and DKEFS BDI	Changes in apathy scores. Apathy correlates with verbal fluency but not with depressive symptoms
Lillo et al., 2011	92 pts	CS	Self-report measures of motor function and mood CBI-R in 81 pts	Reduced motivation: 80 % (apathy in 41 %). Stereotypical and abnormal motor behaviors: 20 %; FTD: 11 %
Lillo et al., 2012	140 CG	CS	CG burden: Zarit Burden Interview CG mood: DASS, Pts behavioral changes: CBI-R	Behavioral changes in 10-40% of pts; Depression, anxiety in 20% of CG; high burden in 48% of CG; Strongest predictor of high CG burden = pts’ abnormal behavior
Marconi et al., 2012	10 pts with tracheostomy and their CG	CS	Anxiety and depression with the HADS Personality of CG using the Big Five Questionnaire (BFQ)	A trend of aggression and high level of obsessiveness in ALS pts. High levels of anxiety in both pts and CG. Higher scores in the dimension of conscientiousness in CG
Mioshi et al., 2014	219 pts 20 pts at 6m	Co LS	MiND-B ALS-FRS	Neuropsychiatric symptoms appear before classic motor features. Not associated with survival. No significant change at 6 mo
Murphy J. M. et al., 2007	23 pts	CS	Neuropsychological and neurobehavioral assessment : ALS-CBS	No impairment: 11 pts; behavioral changes: 4; FTD: 5;other :3 (Alzheimer : 1)
Ohta et al., 2017	57 ALS, 5 ALS-FTD, 12 FTD, 35 NC	CS	Cognitive, behavioral, affective and activities of daily living assessment	FAB and MoCA useful to assess frontal cognitive impairments. ALS-FTD-Q useful to detect mild behavioral and affective disturbances.
Poletti et al., 2018	168 pts at BL 48 after 6 mo 18 after 12 mo 5 after 24 mo	LS	ECAS, FAB and MoCA BDI and STAI/Y	No cognitive deterioration across follow-ups. improvement of some ECAS scores over time due to possible practice effects. Apathy/Inertia = most common behavioral symptom, but no worsening over time.
Rabkin et al., 2016	247 pts	CS	Cognition-behavior : CBS Psychological : PHQ	40 % ci, 9% bi, 18% ci and bi, 12 % Major or minor depression; 12% Bi associated with depression
Radakovic et al., 2017	30 ALS pts / 29 NC	CS	Apathy subtypes with the self- and informant/carer-rated DAS, Cognition: ECAS Comprehensive neuropsychological battery	Increased Initiation apathy was the only significantly elevated subtype in ALS. Initiation apathy associated with verbal fluency deficit, and Emotional apathy, with emotional recognition deficits
Terada et al., 2011	24 pts	CS	Behavioral changes: the FrSBe, ALSFRS respiratory function, arterial blood gases	No correlation between FrSBe scores and ALSFRS, respiratory function, or arterial blood gases. Most frequent behavioral change: apathy
Tremolizzo et al., 2016	84 pts & CG	CS	Pts: ALSCBS-ci and –bi, FAB and BDI CG: BDI and CGBI.	CG burden correlates with pts behavioral but not cognitive changes. CG Burden correlates to CG depression
Unglik et al., 2018	152 pts	CS	EPN-31 (emotional feeling); HADS, The Marin’s apathy evaluation scale Cognitive assessment: ALS-CBS scale.	Apathy: 42 %; related to negative emotions and negatively correlated to cognitive functioning and survival
Witgert et al., 2010	225 pts	CS	FrSBe, Comprehensive neuropsychological evaluation	Changes in the total score: 24.4% (firstly apathy). Cognitively impaired pts have worse total and apathy scores. Behavioral changes in 16 % of cognitively intact pts
Woolley et al., 2010	17 ALS 4 ALS-FTD	CS	Behavioral changes: FrSBe Pts’ awareness of their behavioral changes	Not demented ALS pts have normal insight compared to FTD-ALS pts who have behavioral changes and no insight
Woolley et al., 2011	24 pts; 24 NC	CS	Apathy Brain imaging: DTI	Apathy correlated to FA in right anterior cingulum; not correlated with disease duration or respiratory dysfunction
Woolley et al., 2018	294 at BL 134 at 12 mo	LS	ALS-CBS, Verbal Fluency Index, Controlled Oral Word Association Test and FBI-ALS	No cognitive decline over time; Behavioral change, with increased disinhibition among patients with abnormal BL behavioral scores; BL behavioral problems associated with advanced, rapidly progressive disease

*AES, Apathy Evaluation Scale; ALS-FRS, ALS Functional Rating Scale; BBI, Beaumont Behavioral Inventory;CBI, Cambridge Behavioral Inventory; CGBI, Caregiver Burden Inventory; DAS, Dimensional Apathy Scale; DASS, Depression, Anxiety and Stress Scale; DKEFS, Delis-Kaplan Executive Functioning Scales; EPN-31, Positive and Negative Emotionality Scale; HADS, Hospital Anxiety and Depression Scale; MND, Motor Neuron Disease; MoCA, Montreal Cognitive Assessment; PWI, Personal Wellbeing Index; WM, White Matter. For more please refer to previous tables.*

Apathy seems to be the most common behavioral change (Grossman et al., 2007; Witgert et al., 2010; Lillo et al., 2011; Kasper et al., 2015). The most frequently encountered subtype of apathy in ALS is lack of initiation, that is, a lack of motivation to self-generate thoughts (Radakovic et al., 2017). Patients in the advanced stage may display aggressiveness and obsessiveness (Marconi et al., 2012). Disinhibition, impulsivity, lack of foresight and planning, distractibility, reduced concern for hygiene, irritability, increased self-centeredness and reduced concern for the feelings and needs of others, new unusual habits, loss of insight, and blunting of the primary emotions of happiness, sadness, fear and anger have also been reported (Gibbons et al., 2008; Giordana et al., 2011; Burke et al., 2017). Finally, changes such as aspontaneity, disorganization, and mental rigidity have also been observed (Consonni et al., 2013). Insight on behavioral changes is altered in ALS-FTD, but not in ALS without dementia (Woolley et al., 2010).

##### Implications for the disease course

The presence of behavioral symptoms has practical implications, as it impacts the patients’ psychological state (and that of their caregivers), with consequences for their QoL and indeed their prognosis.

Rabkin et al. (2016) showed that whereas cognitive status does not correlate with mood, patients with behavioral impairment report more depressive symptoms, greater hopelessness, negative mood, and more negative feedback from spouses or caregivers. Caga et al. (2018) found that apathy was associated with more depressive symptoms and a poorer QoL, especially for achievement in life and community connectedness. Aggressiveness and obsession in the advanced stages of the disease correlate with a high level of anxiety in both patients and caregivers (Marconi et al., 2012). Unglik et al. (2018) reported that both apathetic and nonapathetic patients reported anxious and depressive symptoms, and the only significant difference between the two groups was that apathetic and anxious patients experienced more negative emotions, including sadness, shame and anger, than anxious patients without apathy.

The strongest predictor of high caregiver burden is patients’ abnormal behavior (e.g., apathy and disinhibition), rather than physical disability (Lillo et al., 2012; Watermeyer et al., 2015; Tremolizzo et al., 2016). The level of depressive and anxious symptoms in caregivers is also correlated with behavioral changes (Watermeyer et al., 2015; Tremolizzo et al., 2016). Caregiver burden is further influenced by patients’ everyday skills, motivation and memory, mostly because poor motivation, memory dysfunction, and difficulty performing activities of daily living require more support in the shape of direct supervision, prompting, or hands-on care (Andrews et al., 2017b).

Survival in ALS is highly influenced by the presence or absence of apathy, with a median survival time of 21.7 months in the case of moderate-to-severe apathy, 46.9 months in the case of mild apathy, and 51.9 months when apathy is absent (Caga et al., 2018). Apathy correlates negatively with survival time (Unglik et al., 2018).

##### Associated factors

Behavioral changes can occur either on their own or in the presence of a cognitive deficit (Murphy J. M. et al., 2007; Witgert et al., 2010). When cognitive impairment is present, behavioral symptoms seem to be greater (Witgert et al., 2010; Consonni et al., 2013). Apathy has been found to be associated with verbal fluency, leading some authors to suggest that apathy in ALS is underpinned by the medial prefrontal cortex (Grossman et al., 2007; Radakovic et al., 2017). Although Burke et al. (2017) found that social cognitive performances predicted behavioral changes, others have failed to do so (Terada et al., 2011).

Studies assessing relationships between behavioral changes and physical parameters have yielded somewhat conflicting results. Terada et al. (2011) found that apathy was correlated with the ALS condition *per se*, and not with the physical disability. Their population, however, consisted of only mildly disabled patients, who needed no assistance with activities of daily living, and all had normal blood gases. By contrast, in a larger sample of patients, with a wider range of physical impairments (although no details were given about their respiratory status), Ohta et al. (2017) did find a correlation between scores on the ALS-FTD Questionnaire and ALS Functional Rating Scale. Whereas the motor deficit may not directly impact behavior in mildly impaired patients, it is obviously mandatory to control for physical parameters, and first and foremost for blood gases, to avoid erroneously ascribing the consequences of hypercapnic encephalopathy to the cerebral neuronal involvement of ALS in the advanced stage.

Bock et al. (2017) found no association between bulbar or spinal onset and behavioral changes at baseline, whereas other authors (Gibbons et al., 2008; Crockford et al., 2018) have reported that bulbar palsy is associated with a higher rate of behavioral change.

#### Over Time

The way behavioral symptoms change as the disease progresses is still a matter of debate.

Most cross-sectional studies have shown no correlation between the severity of behavioral changes and time elapsed between disease onset and the time of study, suggesting that there is no significant decline over time (Woolley et al., 2011; Bock et al., 2017; Femiano et al., 2018). Crockford et al. (2018) assessed behavior in a sample of 149 patients using the ECAS caregiver behavioral interview and examined whether behavior was related to disease stages according to King’s Clinical Staging System (stage 1 from stage 4 depending on the number of affected bodily regions). Almost 40% of patients were behaviorally impaired (most of them with apathy) and a higher number of behavioral features was found across advancing stages. This result suggests that, contrary to what had been reported previously, behavioral and cognitive impairments are more severe in more severe disease stages.

Similarly, most of the longitudinal studies support the idea that behavioral manifestations over time may either increase (Bock et al., 2017) or appear as the illness progresses, with the emergence of frustration tolerance, reduced insight, mental rigidity, and lack of interest (Bock et al., 2017; Woolley et al., 2018). The severity of these disturbances increases faster over time when dementia is present in patients with the C9+ mutation (Floeter et al., 2017). Poletti et al. (2018) found no change over time in the prevalence of behavioral changes as measured with the ECAS, but did find an increase in behavioral disturbances as measured with the Frontal Behavioral Inventory, which is possibly a more sensitive tool, as it quantifies patients’ performances, whereas the ECAS only indicates whether or not there are changes.

#### Neuroimaging Correlates

Most studies addressing the issue of the neural correlates of behavioral changes in ALS point to a significant correlation between apathy scores and prefrontal cortex atrophy, especially in the orbitofrontal and dorsolateral areas (Tsujimoto et al., 2011; Consonni et al., 2018), whereas disinhibition is negatively correlated with thickness of the right frontotemporal and cingulate cortices (Consonni et al., 2018).

Diffusion tensor imaging studies have shown a significant negative correlation between apathy scores and fractional anisotropy in the right anterior cingulate region, corpus callosum, bilateral amygdalae, left thalamus, and fornix, with atrophy of these brain regions (Woolley et al., 2011; Branco et al., 2018; Femiano et al., 2018). Correlations between apathy and the prefrontal cortex have also been found in other neurological diseases.

On the whole, the relationships between the anterior cingulate (and possibly some subcortical structures) and apathy, and between the anterior temporal lobe and disinhibition, appear quite consistent across studies.

## Psychological Adjustment (Table 4)

### Psychological Reactions and Wellbeing

#### Baseline

Chronic diseases induce a wide range of psychological responses, such as uncertainty about the future, anxiety, and depression. These psychological responses can have a major impact on health, through for example the perceived somatic symptom burden, adherence to treatment and compliance with care, malnutrition, and mortality (Cukor et al., 2006; Katon et al., 2007), and therefore need to be detected and supported.

**TABLE 4:** Psychosocial adjustment and coping in ALS.

**Authors/year**	**Number of pts**	**Type of study**	**Outcomes measures**	**Main findings**
Albert et al., 2005	53 pts	CS	Prevalence of wish to die and its determinants	18.9% express the wish to die. More likely to have depression, less optimism, less comfort in religion, and greater hopelessness. 5.7% having hastened dying reported reduction in suffering in the final weeks of life
Atassi et al., 2011	127 pts	CS	Depression: ADI-12	29% moderate or severe depression, not correlated to disease duration
Brown and Mueller, 1970	10 pts/controls with chronic diseases	CS	IECS MAACL MMPI	Active masterful behavior. Exclusion of dysphoric affect from awareness. Independence and competent behavior
Bungener et al., 2005	27 pts	CS	DSM-IV, Covi anxiety scale, MADRS, Depressive Mood Scale	No severe depression or anxiety. Emotional reactions in the first 6 months after diagnostic disclosure
Caga et al., 2015	27 pts	CS	DASS-21	Lengthy diagnostic interval / higher depressive symptoms
Chen et al., 2015	93 pts and CG	CS	Depression and anxiety: Hamilton depression and anxiety scales	Depression and anxiety rates in pts correlate with CG but not with disease duration or physical incapacity
Chiò et al., 2005	60 pts / 60 CG	CS	CG burden: CBI QoL: MQoL Depression: ZDS Perceived Burden: SPBS	Depression: 18% pts,7% CG CG burden // CG’s mood and pt’s physical disability. Depression of CG/pt correlate
Cui et al., 2015	100 pts / 100 NC		Cognition: MMSE Anxiety: SAS Depression: SDS Functional state: ALSFRS	MMSE negatively correlated with disease duration and ALS-FRS. Higher depression and anxiety in pts than in NC
De Groot et al., 2007	73 pts / general population	Prospective cohort study BL, 6 & 12 mo	Measure of QoL: SF-36 Functional disability: ALS-FRS	QoL lower than controls (Physical Functioning, Role Physical, Social Functioning) but stable over time
Fang et al., 2008	6642 pts	Population-based cohort study	Suicide rate in ALS pts / general population	Suicide risk 6 x in ALS pts. Higher in younger pts and 1^st^ year after 1^st^ hospital stay
Ferentinos et al., 2011	37	CS	Depression: SCID-IV, BDI, HADS, ADI-12 and CES-D	21-25% major depression with SCID, CES-D and BDI
Ganzini et al., 1998	100 pts / CG	Prospective cohort	Determination of pts and caregivers’ attitude toward assisted suicide	56% would consider suicide. Men, higher education, less religiosity, higher scores for hopelessness, lower QoL increase positive attitude towards assisted suicide 73%: pts and caregivers have the same point of view
Gauthier et al., 2007	31 pts / 31 CG	LS: BL – 9 mo	Depression: ZDS QoL: (MQoL) Caregivers’ burden: CBI Perceived burden: SPBS	Depression and QoL stable over time in pts but Depression and burden increase in CG
Goldstein et al., 2006b	50 CG at BL, 21 on follow-up	LS BL-follow-up 6 mo intervals	Mood, burden and strain, Social support and marital relationship	Main predictor of distress in ALS pts CG over time is poor social support
Goldstein et al., 2006a	50 at BL 26 over time	LS: BL, 6 and 11 mo	Predictors of psychological distress	Affective state and self-esteem predicted by social support and pre-illness marital intimacy
Goldstein et al., 1998	19 pts / 19 CG	CS	Psychological distress in pts and CG and their determinants	In pts: anxiety and depression correlate to physical disability In CG: distress depends on pts’ funct. impairment, and intimacy loss. Perceived good social support correlates to future ability to cope
Grehl et al., 2011	41 pts / 41 relatives	CS	Depression with ADL-12; QoL with MLDL in pts and relatives	Mood and QoL correlate between pts and relatives but not to functional impairment
Hammer et al., 2008	39 pts	CS	Assessment of depression by DSMIV, BDI and ADI-12 scales	10 % depressed by SCID ADI-12 recommended for screening depression in ALS
Hillemacher et al., 2004	41 pts	CS	Depressive symptoms Correlation to the ALS-FRS, disease duration, age and sex	Depression correlated with swallowing and breathing but not with age, sex or ALS-FRS; depression correlated with duration
Jakobsson Larsson et al., 2016	36 pts	LS: BL and at 5 time over a period of 2 years	Coping strategies: with Motor Neuron Disease Coping Scale Well-being: Hospital Anxiety and Depression Scale Physical abilities	No changes over time in coping strategies; Psychological state correlates with some coping items (e.g. negative correlation with depressive symptoms and “positive action, positive thinking and independence”)
Jakobsson Larsson et al., 2017	36 pts	Longitudinal with a follow up periode of 2 years	QoL: SEIQoL-DW; Emotional distress: HADS	Anxiety 11%, depression 5% early on after diagnosis Anxiety decreases over time QoL related to depression soon after diagnosis
Jelsone-Swain et al., 2012	22 pts / 17 NC	CS	Neuropsychological assessement Depression: GDS, BDI	Cognitive tests: Pts < NC. No influence of depression. Depression correlated with limb function
Houpt et al., 1977	40 pts	CS	Patient’s control: IECS; Depression: BDI, MAACL Denial	Depression 22%. Dysphoria frequently found. No specific use of denial or internal locus of control
Lillo et al., 2012	140 CG	CS	CG burden: Zarit Burden Interview CG mood: DASS-21 Pts; behavior: CBI-R	Behavioral changes in 10–40% of pts; Depression, anxiety in 20% of CG; high burden in 48% of CG; Strongest predictor of high CG burden = pts’ abnormal behavior
Lou et al., 2003	25 ALS / 22 NC	CS	Fatigue and depression: MQoL CES-D	Fatigue and depression higher in pts/NC Associated with poorer QoL
Lulé et al., 2008	1: 39 pts 2: 30 pts / 30 NC	CS: pts / NC LS: BL and 80 - 100 days later	Depression with ADI-12; QoL with SEIQoL-DW	Depression 28% not correlated to physical impairment QoL = NC and not correlated to physical impairment
Lulé et al., 2012	30 ALS pts 29 cancer pts 29 NC	CS	Depression: BDI; QoL: SEIQoL-DW Coping strategy: Jerusalem Coping scale	Good psychosocial adjustment and subjective QoL in both patient groups
Matuz et al., 2010	27 pts	CS	Depression, QoL Predictors: social support, cognitive appraisal, coping strategies	Perceived social support predicts depression and QoL. Appraisal of coping potential predicts depression. No impact of physical status
Matuz et al., 2015	27 pts	Longitudinal with four evaluation in 2 years	QoL; Depression Social support, cognitive appraisals, and coping strategies	Social support, cognitive appraisals, coping strategies are the best predictors of QoL and depression
McElhiney et al., 2009	223 at BL 113 at 3 mo 65 final visit	LS	Fatigue and depression prevalence at BL, 3 and 6 mo y PHQ-9 interview	Fatigue associated to severity and more prevalent and persistent than depression
McElhiney et al., 2014	81 pts / 81 CG	LS: BL, 3 mo, 6 mo	ALS-FRS, QoL and Goal Assessment Scale (GAS)	QoL, GAS: no consistent correlations with ALSFRS-R change
Miglioretti et al., 2008	74 pts	CS	ALS -FRS Illness representation: common sense model	QoL, mood, and illness representation correlate with functional state and respiratory capacity.
Rabkin et al., 2000	56 pts, 31 CG	LS BL / 3-8 mo	Pts: DSM-IV, BDI, STAI, QoL, outlook about future and ZARIT caregiver burden	Pts: major depression 2% by DSM-IV and 28% by BDI. Psychological distress not related to illness progression CG: low rate of depression but high perceived burden
Rabkin et al., 2005	80 pts BL / 61 pts follow-up	LS / monthly for 15 mo	Prevalence of depression over time: PHQ and BDI	20% depression increasing to 31% before death
Rabkin et al., 2009	71 pts / 71 CG	LS: BL and monthly for 51 mo	Depressive symptoms, DSM-IV disorders, Coping strategies Caregiver burden satisfaction with care-giving	CG burden & depression // Reliance on avoidance, perceived burden, fatigue, feeling that pt critical and unappreciative; long-term mechanical ventilation; pts’ plans and supportiveness
Rabkin et al., 2015	329 pts	CS	Prevalence of depression and wish to die at BL	Depression 12%, related to ALSFRS and motor strength. Wish to die 19% but only 1/3 of which depressed
Rabkin et al., 2016	247 pts	CS	Cognitive, behavioral or, mood impairment by CBS and PHQ9	Cognitive impairment:40 %; Behavioral impairment:9%; Both:18%; depression:12 % Behavioral impairment associated to depression
Roach et al., 2009	55 pts / 53 CG	LS	QoL: MQoL	Pt’s Qol: no change over time Total QoL and QoL related to physical symptoms decline in CGs; younger CG = lower QoL
Roos et al., 2016	1752 ALS pts and 8760 NC	R	Depression: ICD-10 and use of antidepressants	Higher risk of depression the year before and the year after the diagnosis of ALS
Sandstedt et al., 2016	60 pts	CS	HRQL/disease severity, fatigue, anxiety, depression, social activities, coping and mechanical ventilation	Severe disease, weak coping capacity, fatigue, mechanical ventilator and anxiety and/or depression associated with worse HRQL
Siciliano et al., 2017	96 CG	CG	Burden, depression and anxiety Coping strategy: CISS Pts’ cognition/behavior	Burden, anxiety, depression in CG related to: emotion-oriented coping strategy and Pts’ functional dependence
Taylor et al., 2010	51 ALS pts 39 other neuromuscular disorders		Depression: BDI, HADS and MDI	Same depression rates in both groups
Thakore and Pioro, 2016	964	Retrospective cohort	Depression: PHQ-9 and its associated factor	Depression 49 %. High PHQ-9 scores predict mortality. PHQ-9 correlates with QoL. Depression correlated with pseudobulbar symptoms and advanced disease
Turner et al., 2016	National psychiatric database / reference cohort	R	Evaluation of the risk to develop ALS in psychiatric pts	Psychiatric disease, especially bipolar disorder and schizophrenia = higher risk to develop ALS, mainly the year after psychiatric illness onset
Verschueren et al., 2018	71 pts	Prospective, observational cohort study	Depression: BDI Columbia Suicide severity rating scale Reasons for Living inventory for adults.	39% express either passive or active suicidal ideation. Depressive symptoms, worse disability and coping beliefs scores more present in pts expressing suicidal ideation
Vignola et al., 2008	75 pts / CG	LS	Depression: ZDS Anxiety: STAI	High anxiety in pts and CG during the diagnostic phase QoL decreases in CG but not in pts at follow-up
Wei et al., 2016	91 pts	CS	Neuropsychiatric symptoms and cognition: NPI, ACE-R, FAB	Depression 59%, anxiety 41%, lability 26%. NPI correlates with ACE-R but not with FAB
Wicks et al., 2007	104	CS	Depression: BDI, HADS Anxiety: STAi	Depression: 54% with BDI, 25% with HADS Anxiety 35% state 8% trait

*ADI, Assessment of Depression Inventory; CES-D, Center For Epidemiologic Studies Depression Scale; CISS, Coping Inventory For Stressful Situations; DSM, Diagnostic and Statistical Manual of Mental Disorders; GDS, Global Deterioration Scale; ICD-10, International Classification of Diseases; IECS, Internal-External Control Scale; HRQL, Health-Related Quality of Life; MAACL, Multiple Affect Adjective Check List; MLDL, Munich Quality-of-Life Dimensions List; MMSE, Mini-Mental State Examination; MQoL, Mcgill Quality-of-Life Questionnaire; SAS, Self-Rating Anxiety Scale; SCID, Structured Clinical Interview For DSM-IV; SDS, Zung Self-Rating Depression Scale; SeiQoL-DW, Schedule For The Evaluation of Individual Quality of Life; SPBS, Self-Perceived Burden Scale; ZDS, Zung Depression Scale. For more please refer to previous tables.*

Patients with ALS were initially described as *abnormally positive* (Brown and Mueller, 1970) and, in contrast to what is usually described in other serious chronic somatic diseases, had a relatively low prevalence of depressive disorders ranging from 0% (Rabkin et al., 2000; Bungener et al., 2005) to 10% (Hammer et al., 2008; McElhiney et al., 2009; Rabkin et al., 2016; Wei et al., 2016), according to their responses to semi-structured questionnaires based on international criteria for depression.

Nevertheless, these results have to be considered in the light of several additional factors. First, when patients are assessed at the extreme stages of the disease (either soon after diagnosis or at a very advanced stage), validated scales indicate that the prevalence of depressive symptoms is around 20% (Rabkin et al., 2005), and this figure rises to above 50% when self-report questionnaires are used (Wicks et al., 2007). Second, a history of depression before the onset of ALS increases the prevalence rate from 10 to 21% when patients are assessed with a semi-structured questionnaire (McElhiney et al., 2009; Ferentinos et al., 2011). An interesting study conducted by Roos et al. (2016) found that the risk of receiving a diagnosis of depression was increased during the year before and the year after the diagnosis of ALS. This is also true for other major psychiatric disorders, namely schizophrenia, bipolar disorder, and anxiety, some of which may predate the diagnosis of ALS by as much as 5 years (Turner et al., 2016). Self-report questionnaires designed to probe depressive symptoms may have their limits, insofar as they are not able to diagnose a depressive state with certainty, leading to potential overestimation of the rate of depression, but they do have the advantage of detecting the potential presence of depressive symptoms that reflect a degree of distress. Studies using this type of instrument report higher rates of depression of above 30% (Lulé et al., 2008; Atassi et al., 2011; Grehl et al., 2011; Carvalho et al., 2016). Thus, even when the official diagnostic criteria are not met, it does not mean that patients do not feel they are affected by their disease. One must thus be aware that depression rates in ALS vary greatly, depending on the tools used to assess it. Finally, studies comparing the rate of depression in patients with ALS versus other chronically and seriously ill patients (e.g., with neuromuscular disease or receiving palliative care for cancer) have failed to find any significant differences, with 8–10% of patients in each group having a diagnosis of major depression according to DSM-IV criteria, and 50% mild-to-moderate depressive symptoms, as measured with the Beck Depressive Inventory (Taylor et al., 2010; Lulé et al., 2012).

Furthermore, more than one third of patients with ALS are on antidepressants (Pisa et al., 2015). Not all studies assessing the prevalence of depression in ALS take this fact into account, which could induce a bias and result in underestimation of the prevalence of depression in this population.

Intuitively, one might assume that the worsening of physical disability increases signs of depression. However, many studies have found either no such link or an inverse link between the severity of the motor disability and scores on mood scales (Lulé et al., 2008; Jelsone-Swain et al., 2012; Chen et al., 2015; Thakore and Pioro, 2016; Wei et al., 2016). This is only true, however, for the consequences of spinal involvement, as the presence of bulbar symptoms, or of breathing difficulty, does increase depressive symptoms (Hillemacher et al., 2004; Goldstein et al., 2006a; Miglioretti et al., 2008; Jelsone-Swain et al., 2012).

Studies looking for a link between disease duration and the severity of depressive symptoms have reported contradictory results, with some finding a positive correlation, some a negative one (Cui et al., 2015; Hillemacher et al., 2004, and others no link at all (Atassi et al., 2011; Caga et al., 2015).

Severe somatic diseases may induce a number of other psychological reactions, such as anxiety, hopelessness, or suicidal thoughts. Kurt et al. (2007) found that the prevalence of anxiety in ALS ranged from 0 to 30%. Vignola et al. (2008) reported that almost 75% of patients experienced moderate-to-severe state anxiety at baseline, which was correlated with trait anxiety. However, Pagnini et al. (2012) found that only 20% of patients had scores above the anxiety cut off. Suicidal thoughts are not rare in patients, ranging from 19 to 39% across studies (Albert et al., 2005; Rabkin et al., 2015; Verschueren et al., 2018). This rate rises to more than 50% when assisted suicide is considered (Ganzini et al., 1998). Patients have an almost 6-fold higher risk of suicide, especially in the first year after symptom onset and when they are younger (Fang et al., 2008). A wish to die is not always associated with a depressive state (Albert et al., 2005) but it is linked to less optimism, less comfort in religion, and greater hopelessness (Albert et al., 2005; Verschueren et al., 2018).

In addition to psychological signs of distress such as depression or anxiety, the estimation of wellbeing is based on the person’s satisfaction with his or her QoL. In patients with ALS, QoL is found to be high, especially when the measurement scales are adapted to the disease (Norris et al., 2010; Jakobsson Larsson et al., 2017) and avoid lending too much importance to the patient’s physical state (Norris et al., 2010; Jakobsson Larsson et al., 2017). The Amyotrophic Lateral Sclerosis Quality of Life (ALSSQOL) was specifically designed for ALS, and has a revised and a short form (Felgoise et al., 2018) that give a balanced appraisal. Qol depends not only on patients’ physical disabilities, but also on their religiosity/spirituality and sociability (Norris et al., 2010; Simmons, 2015). Other factors influencing QoL are anxiety and depression, the ability to cope with physical disabilities, fatigue, and hopelessness (Lou et al., 2003; Abe, 2004; Pagnini et al., 2012; Sandstedt et al., 2016; Jakobsson Larsson et al., 2017).

#### Over Time

Surprisingly, longitudinal studies of changes in patients’ mood as the disease progresses report either stability (Rabkin et al., 2005; Gauthier et al., 2007; McElhiney et al., 2009; Matuz et al., 2015) or a decrease in the depression rate (McElhiney et al., 2014). When Goldstein et al. (2006a) assessed psychosocial factors influencing patients’ psychological state (level of depression and anxiety) at baseline and 6 and 11 months later, they found that the quality of premarital intimacy and social support at disease onset influenced the psychological wellbeing of patients in the more advanced stages of the disease. Anxiety is particularly high in the diagnostic phase, but tends to decrease thereafter in patients, though not in caregivers (Vignola et al., 2008). Importantly, these authors also found that in caregivers, state anxiety was linked to trait anxiety, whereas in patients, it was correlated with clinical features such as a shorter disease course and the presence of depression. Together with other factors, anxiety negatively impacts QoL (Jakobsson Larsson et al., 2017).

Caregivers’ psychological distress and perceived burden is often reported to increase over time (Goldstein et al., 2006b; Gauthier et al., 2007). The burden is determined by a combination of factors, including both the caregiver’s own personality and the patient’s characteristics. Regarding the latter, Lillo et al. (2012) found that behavioral changes are a greater determinant of caregivers’ wellbeing than physical disability, although motor impairment also plays a part, according to Chiò et al. (2005). Behavioral changes may include apathy, loss of empathy, and a lack of appreciation of the efforts made to satisfy their needs (Rabkin et al., 2009). As the disease progresses, patients’ plans for future, particularly regarding life support, may also play a part and influence their caregivers’ QoL. A longitudinal study by Rabkin et al. (2009) found that caregivers tended to be less depressed as the disease advanced, regardless of the outcome (death or tracheostomy), whereas the depression scores of patients remained stable.

Quality of life as reported by patients remains stable even in the advanced stages of the disease (Rabkin et al., 2000; De Groot et al., 2007; Roach et al., 2009). Religiosity, social support, level of anxiety and depression remain the mean determinants of wellbeing, despite the increase in motor impairment (Jakobsson Larsson et al., 2017).

### Adaptive Mechanisms in the Face of the Disease

To explain the fact that patients with ALS have a relatively good adaptive reaction to their illness, some authors have suggested that they are in denial of the disease, thus protecting them from depression (Brown and Mueller, 1970; Miglioretti et al., 2008), but this hypothesis has not been confirmed (Houpt et al., 1977).

*Psychological adjustment* refers to the psychological processes that take place in response to a stressful situation like chronic illness and associated treatment. Various models have been developed, including the stress coping model (Lazarus and Folkman, 1984), illness representation model, adaptive tasks and coping model (Moos and Holahan, 2007), and adjustment model (Moss-Morris, 2013). Based on Lazarus and Folkman’s theoretical framework, Matuz et al. (2010) developed an interesting integrative model of patients’ adaptation in the face of the disease, whereby patients’ mood state and QoL are influenced by social support, cognitive appraisal and coping strategies. The latter include problem management, problem appraisal, emotion regulation, and emotional avoidance. *Social support* encompasses perceived social support, received social support, and need for social support. Matuz et al. (2010) found that patients used emotion regulation and looked for social support, and the more they used emotion-focused strategies, the lower they scored on a depression scale. When Lulé et al. (2012) compared patients with ALS and patients with cancer on psychological adjustment, they failed to find any significant differences in the use of coping strategies, even if, on average, the ALS group scored lower than the cancer group on a scale measuring active coping strategies (thinking about the situation and trying to solve it, taking an adequate step to deal with their condition). By contrast, depressive symptoms and a high level of burden are more often present in caregivers if they use emotion-focused coping strategies (Siciliano et al., 2017). Factors that have been shown to help caregivers cope with the impact of the disease include social support, as well as anticipatory coping with foreseeable difficulties (Goldstein et al., 1998).

Studies addressing changes in psychological adjustment strategies in ALS over time have yielded conflicting results. Jakobsson Larsson et al. (2016) found that the patients used the same coping strategies throughout the disease course, namely support seeking, positive action, independence, and positive thinking. By contrast, Matuz et al. (2015) reported that while patients used both problem- and emotion-focused coping strategies at disease onset, they used less emotion regulation later on. On the other hand, they continued to have higher perceived social support and an accurate assessment of their own coping potential. These two factors were correlated with depressive scores at follow up. *Perceived social support*, which reflects patients’ view about the amount and quality of support they receive, can help patients use effective coping strategies, encourage positive health behaviors, and reduce physiological reactivity to stress. A positive subjective appraisal of one’s coping potential indicates that patients feel they are keeping control over their state, which probably decreases their anxiety over physical loss.

At a time when some people advocate legalizing euthanasia for intractable disease, accurate knowledge of the mechanisms by which many patients with ALS succeed in coping with such a dreadful disease is crucial, and could greatly enhance the assistance given to both patients and caregivers in their daily struggle.

## Conclusion

The objective of this review was to provide readers with a clear picture of all the manifestations of ALS both at baseline and throughout the course of the disease. Unlike the relentless physical deterioration, cognitive, behavioral and psychological changes are extremely variable. The heterogeneity of clinical situations, and in particular the variety of symptoms present at baseline, seems to strongly influence patients’ clinical course. Regarding their psychological reactions, even if they rarely develop a severe psychiatric illness, they can experience considerable distress, especially at the time of diagnosis and in the advanced stage of the disease. Caution must be taken not to minimize these psychological reactions and to give patients the best possible personalized support at these times. A careful examination of the psychological trajectory shows that the increase in motor disability is not the only determinant of psychological wellbeing. The quality of social support and the use of appropriate adaptive strategies enable patients to cope with their condition and to maintain good psychosocial functioning as the disease progresses.

This review also illustrates the theoretical and practical difficulties that may arise when investigating cognitive, behavioral and psychological aspects of ALS. The first concerns the tools that are to be used. First, these must be adapted to the physical disability of patients with ALS. Potentially confounding variables, such as respiratory insufficiency, have to be controlled. As we have seen, a large number of tests have been used by the different authors. Such a diversity makes comparisons difficult and may at least partially account for the discrepancies that are often observed across studies. Investigators will have eventually to try to agree about which tests should be used, either as routine tests (such as the ECAS for the cognitive assessment, or ALS-FRS or the bulbar Norris scale for physical parameters) or for more specific purposes. It will also be interesting to further study less extensively explored cognitive domains such as memory or social cognition.

Another difficulty is that of longitudinal studies. These are particularly challenging, because of the major physical changes that occur over time. It is not clearly known, for example, whether a cognitive impairment, if any, worsens in line with the physical disability or not. Also, the short life expectancy of many patients is responsible for a substantial drop-out rate. These difficulties can be addressed by recruiting as many patients as possible, preferably in the setting of ALS centers. Bigger samples may also help to tackle the problem of physical and neuropsychological heterogeneity. For example, the seemingly simple question as to whether bulbar-onset patients are more cognitively affected than spinal-onset ones or not is still debated. Likewise, there have been very few studies about the relationships between the cognitive (or psychological, or behavioral, for that matter) and physical profiles of patients. Finally, morphological and functional cerebral imaging studies should be increasingly undertaken in order to learn more about the mechanisms of neuropsychological impairment, and to improve as much as possible the care of patients with ALS.

## Author Contributions

BD, SB, and FV contributed to conceptualization and methodology. SB wrote the first draft of the manuscript. BD, FV, and FE reviewed the manuscript. SB, FV, and FE edited the manuscript.

## Conflict of Interest Statement

The authors declare that the research was conducted in the absence of any commercial or financial relationships that could be construed as a potential conflict of interest.
